# Supplementary material for: Determinants of Vitamin D Supplementation among Individuals with Type 1 Diabetes
Source: Int J Environ Res Public Health. 2020 Jan 22;17(3):715. doi: 10.3390/ijerph17030715 (PMC7036832; doi:10.3390/ijerph17030715)
Supplement: Supplementary file 1 [file ijerph-17-00715-s001.pdf]

Supplementary

Table S1.
The authors questionnaire on Vitamin D supplementation among individuals with diabetes.

Polish version	English version
1.Proszę podać swoją płeć:<br>□ kobieta<br>□ mężczyzna	1.Please mark your gender:<br>□ female<br>□ male
2. Proszę wpisać typ cukrzycy na którą Pan/Pani Cukrzyca choruje?<br>□ Cukrzyca typu 1*<br>□ Cukrzyca typu 2<br>□ Cukrzyca typu 3**<br>□ Nie choruję na cukrzycę<br>*(w tym LADA)<br>**(inne powody cukrzycy np. przewlekłe zapalenie)	2.Please mark the diabetes type that applies to you:<br>□ Type 1 Diabetes*<br>□ Type 2 Diabetes<br>□ Type 3 Diabetes**<br>□ I do not suffer from diabetes<br>*(including LADA)<br>**(other reasons of diabetes e.g. chronic inflammation)
3. Ile ma Pan/Pani lat [w latach, numer]	3. How old are you? [number of years]
4. Jak długo Pan/Pani choruje na cukrzycę [w latach, numer]	4. For how long have you suffered from a diabetes? [number of years]
5. Proszę podać swoją obecną masę [w kilogramach, numer]<br>6. Proszę podać swój wzrost [w centymetrach, numer]	5. Please enter your current weight [number of kilograms]<br>6. Please enter your height [numer of centimeters]
7. Proszę zaznaczyć miejsce zamieszkania<br>□ Wieś<br>□ Miasto do 50.00 mieszkańców<br>□ Miasto powyżej 50.000 mieszkańców*<br>* (m.in. Gniezno, Kalisz, Konin, Leszno Ostrów Wielkopolski, Piła)	7. Please mark your place of residence<br>□ Village<br>□ A city smaller than 50.000 inhabitants<br>□ A city bigger than 50.000 inhabitants*<br>*(e.g. Gniezno, Kalisz, Konin, Leszno Ostrów Wielkopolski, Piła)
8.Proszę zaznaczyć TAK/NIE.<br>Czy cierpi Pan/Pani na...<br>Retinopatię cukrzycową? TAK/NIE<br>Nefropatię cukrzycową? TAK/NIE<br>Neuropatię cukrzycową? TAK/NIE<br>Zespół stopy cukrzycowej? TAK/NIE<br>Makroangiopatię (choroba wieńcowa, zawał serca, udar mózgu lub chorobę tętnic obwodowych)? TAK/NIE<br>Niedoczynność tarczycy (np. choroba Hashimoto)? TAK/NIE<br>Celiakię (chorobę trzewną)? TAK/NIE<br>Astmę oskrzelową? TAK/NIE	8.Please choose YES/NO.<br>Do you suffer from…<br>Diabetic retinopathy? YES / NO<br>Diabetic nephropathy? YES / NO<br>Diabetic neuropathy? YES / NO<br>Diabetic foot ulcers? YES / NO<br>Macroangiopathy (coronary artery disease, myocardial infarction, brain stroke or peripheral artery disease)? YES/NO<br>Hypothyroidism (e.g. Hashimoto disease)? YES/NO<br>Coeliac disease? YES/NO<br>Asthma? YES/NO

9. Proszę zaznaczyć TAK/NIE
Mój lekarz rodzinny zalecał mi suplementację wit. D
TAK/NIE
Mój lekarz specjalista zalecał mi suplementację wit. D.
TAK/NIE
Farmaceuta zalecał mi suplementację wit. D
TAK/NIE
Osoba z grona rodziny zalecała mi suplementacją wit. D
TAK/NIE
Osoba z grona znajomych zalecała mi suplementację wit. D
TAK/NIE
Dowiedziała/em się o potrzebie suplementacji wit. D z Internetu/mediów/książek
AK/NIE
Wiem, że osoba z kręgu najbliższej rodziny (dzieci, rodzeństwo, rodzice, dziadkowie) suplementuje wit. D
TAK/NIE
Wiem, że osoba z mojego najbliższego kręgu znajomych suplementuje wit. D
TAK/NIE

9. Please choose YES/NO
My family doctor has recommended me the vit D supplementation
YES/NO
My medical specialist has recommended me the vit D supplementation
YES/NO
Pharmacist has recommended me the vit D supplementation YES/NO
My family member has recommended me the vit D supplementation
YES/NO
My friend has recommended me the vit D supplementation
YES/NO
I learned about the need of vit D supplementation from the Internet/media/books
YES/NO
I know that a close family member (kids, siblings, parents, grandparents) takes vit D supplements
YES/NO
I know that a close friend of mine takes vit D supplements.
YES/NO

---

10. Czy suplementuje Pan/Pani witaminę D*?
*zarówno w preparatach izolowanych/z dodatkiem wapnia/ witaminy K/multiwitamina
TAK/NIE

10. Do you take vitamin D supplements*?
*any of: isolated formula/with Calcium added/with vitamin K added/multivitamin formula
YES/NO

Jeśli 10. TAK:

11. Proszę zaznaczyć co **w największym stopniu** skłoniło Pan/Panią do suplementacji wit. D?

[**Proszę zaznaczyć jedną odpowiedź**]!
□ porada lekarza rodzinnego
□ porada lekarza specjalisty
□ porada farmaceuty
□ porada/wpływ osoby z rodziny
□ porada/wpływ osoby z grona znajomych
□ wiedza zdobyta w z Internetu/mediów/książek
□ inne…...

12. Kiedy przyjmuje Pan/Pani witaminę D?
□ przez cały rok
□ od października do kwietnia
□ nieregularnie

If 10. YES:

11. Please choose what motivated you **the most** to begin vit D supplementation

[**Please choose only one answer**]!
□ Recommendation of my family doctor
□ Recommendation of my medical specialist
□ Recommendation of a pharmacist
□ Recommendation/influence of a family member
□ Recommendation/influence of a friend
□ Knowledge acquired from the the Internet/media/books
□ other…..

12. When do you take vitamin D supplements?
□ all year long
□ from October to April
□ irregularly

Jeśli 10. NIE:<br><br>11. Proszę zaznaczyć co **w największym stopniu** sprawia,że nie suplementuje Pan/Pani wit. D?<br><br>[**Proszę zaznaczyć jedną odpowiedź**]!<br>□ nie wiem w ogóle w jakim celu mam suplementować wit. D<br>□ uważam, że suplementacja wit. D ma małe znaczenie dla zdrowia<br>□ znam znaczenie suplementacji wit. D, ale szkoda mi pieniędzy<br><br>□ znam znaczenie suplementacji wit. D, ale nie mam motywacji do rozpoczęcia suplementacji<br>□ inne…..	If 10. NO:<br><br>11. Please mark what is **the main cause** that you do not supplement vit D?<br><br>[**Please choose only one answer!**]<br>□ I don't know why I should take vit D supplements<br>□ I think that vit. D supplementation has little meaning for my health<br>□ I know the importance of vit. D supplementation, but I don't want to spend money on it<br>□ I know the importance of vit. D supplementation, but I/m not motivated enough to start supplementation<br>□ Other…...

Table S2.
List of the Facebook group for individuals with diabetes and the number of completed surveys.

Original name	URL	Number of completed surveys
diabetycy26+	https://www.facebook.com/groups/diabetycy26/	88
Freestyle Libre Polska (Pl)	https://www.facebook.com/groups/639932702829756/	67
CUKRZYCA 24H INFO	https://www.facebook.com/groups/604766892967695/	49
Cukrzyca. Sportowa Sekta.	https://www.facebook.com/groups/621331564551503/	45
CUKRZYCA ǀ Grupa mojacukrzyca.org	https://www.facebook.com/groups/mojacukrzyca/	38
Cukrzyca t1 .... bez paniki !!!	https://www.facebook.com/groups/101573080188497/	29
Cukrzyca Typu 1-życie bez barier i na kolorowo.	https://www.facebook.com/groups/408604312853355/	27
Cukrzyca info (słodki spam)	https://www.facebook.com/groups/614149132026605/	22
DIABETYCY	https://www.facebook.com/groups/275811689144810/	22
Cukrzyca - Oddam/Sprzedam/Kupię/Zamienię	https://www.facebook.com/groups/413531028786785/	20
CUKRZYCA TYPU 1 – ODZYSKAJMY KOLORY ŻYCIA	https://www.facebook.com/groups/ct1odzyskajmykoloryzycia/	19
Wszystko o cukrzycy	https://www.facebook.com/groups/408251949244446/	17
Cukrzyca a orzecznictwo o niepełnosprawności	https://www.facebook.com/groups/541716675957857/	16
Stowarzyszenie Przyjaciół Dzieci i Młodzieży z Cukrzycą w Kaliszu	https://www.facebook.com/groups/393304747372125/	12
Cukrzyca Polska ➤ Grupa Dyskusyjna ➤Forum	https://www.facebook.com/groups/161089677754418/	9
Cukrzyca to nie wyrok.	https://www.facebook.com/groups/1079626195484813/	8
Pompa Paradigm VEO \ 722 & Guardian Conect POLSKA CGM Refundacja	https://www.facebook.com/groups/1367648416673139/	8
Cukrzyca a Pompa insulinowa	https://www.facebook.com/groups/333396326816885/	7
DiabCafe-cukrzyca i życie	https://www.facebook.com/groups/570106823086852/	6
Cukrzycy z Poznania	https://www.facebook.com/groups/485198251866858/	6

Cukrzyca Typu 1 / Typu 2 - HOLISTYCZNIE	https://www.facebook.com/groups/240189219887933/	5
Looped Polska (Cukrzyca w Pętli)	https://www.facebook.com/groups/loopedpolska/	5
Poradnik Młodego Diabetyka	https://www.facebook.com/groups/105342996171291/	4
Dexcom Polska użytkownicy	https://www.facebook.com/groups/1114015185302504/	3
Słodki Kraków	https://www.facebook.com/groups/372467293092919/	3
Zdrowy Diabetyk	https://www.facebook.com/groups/172920596737400/	2
Diabetycy cukrzyca BIAŁYSTOK podlaskie	https://www.facebook.com/groups/diabetycy/	2
Cukrzyca typu 1- inne podejście	https://www.facebook.com/groups/246436075913401/	2
Nightscout Polska (Poland)	https://www.facebook.com/groups/NightscoutPoland/	2
Cukrzyca info	https://www.facebook.com/groups/621331564551503/	2
Cukrzyca t1 i Celiakia	https://www.facebook.com/groups/159245124632262/	2
AndroidAPS Polska (Poland)	https://www.facebook.com/groups/2110352665691964/	1
Cukrzyca typu II. Dobre rady,dobry humor,dobrzy ludzie.	https://www.facebook.com/groups/1714912278807840/	1

Table S3.
The authors questionnaire on Vitamin D supplementation among diabetologists.

Polish version	English version
1. Proszę podać swoją płeć:<br>□ kobieta<br>□ mężczyzna	1.Please enter your gender:<br>□ female<br>□ male
2. Liczba lat praktyki zawodowej<br>[w latach, numer]	2. Number of years of medical practice<br>[number of years]
3. Czy suplementuje Pan/Pani witaminę D*?<br>*zarówno w preparatach izolowanych/z dodatkiem wapnia/ witaminy K/multiwitamina<br><br>TAK/NIE	3. Do you take vitamin D supplements*?<br>*any of: isolated formula/with Calcium added/with vitamin K added/multivitamin formula<br>YES/NO
Jeśli tak:	If yes:
4. Proszę zaznaczyć co **w największym stopniu** skłoniło Pan/Panią do suplementacji wit. D?<br>[**Proszę zaznaczyć jedną odpowiedź**]!<br>□ porada lekarza rodzinnego<br>□ porada lekarza specjalisty<br><br>□ porada farmaceuty<br>□ porada/wpływ osoby z rodziny<br><br>□ porada/wpływ osoby z grona znajomych<br>□ wiedza zdobyta w z Internetu/mediów/ książek<br>□ inne…...	4. Please choose what motivated you **the most** to begin Vit D supplementation<br><br>[**Please choose only one answer**]!<br>□ Recommendation of my family doctor<br>□ Recommendation of my medical specialist<br>□ Recommendation of a pharmacist<br>□ Recommendation/influence of a family member<br>□ Recommendation/influence of a friend<br>□ Knowledge acquired from the Internet/media/books<br>□ other…...
5. Kiedy przyjmuje Pan/Pani witaminę D?<br>□ przez cały rok<br>□ od października do kwietnia<br>□ nieregularnie	5. When do you take vitamin D supplements?<br>□ all year long<br>□ from October to April<br>□ irregularly

6. Z jaką częścią swoich pacjentów rozmawia Pan/Pani Doktor nt. suplementacji wit. D?<br>□ Z przeważającą większością (> 75%)<br>□ Ze znaczną częścią (25-75%)<br>□ Z mniejszością (<25%)<br>□ Nie rozmawiam z pacjentami nt. suplementacji wit. D (0%)	6. With what percentage of your patients do you talk about vitamin D supplementation?<br>□ With most of them (>75%)<br>□ With some of them (25-75%)<br>□ With a few of them (<25%)<br>□ I don't talk with my patients about vitamin D supplementation (0%)
7.Proszę zaznaczyć Prawda (P) / Fałsz (F):<br>a) **P/F** mam zbyt mało czasu aby zalecać suplementację wit. D podczas wizyty<br><br>b) **P/F** uważam, że suplementacja wit. D nie przynosi wymiernych korzyści u moich pacjentów<br>c) **P/F** uważam, że nie ma sensu zalecać suplementacji wit. D gdyż pacjenci nie będą stosowali regularnej suplementacji<br>d) **P/F** nie zalecam suplementacji wit. D, aby nie obciążać pacjenta dodatkowymi kosztami<br><br>e) **P/F** uważam, że z pacjentem nt.o suplementacji wit. D powinien rozmawiać każdy profesjonalista medyczny (zarówno lekarz rodzinny, specjalista, farmaceuta, pielęgniarka itd.)	7. Please choose True (T) or False (F).<br>a) **T/F** I do not have enough time to recommend vitamin D supplementation during a visit<br>b) **T/F** I think that vitamin D supplementation is not significantly beneficial for my patients<br>c) **T/F** I don't think it is worth prescribing vitamin D supplements, because patients will not take them regularly<br>d) **T/F** I do not prescribe vitamin D supplements so as not to create an additional cost for them<br>e) **T/F** I think that each medical professional should talk about vitamin D supplementation with the patient (general practitioners, specialized doctors, pharmacists, nurses etc.)

Table S4.
Comparison of the answers from paper and digital surveys. Data presented as median (interquartile range) or number (percentage).
a) general characteristics and diabetes history

variables	Total n = 734 (100%)	Paper surveys n =184 (25.1%)	Digital surveys n = 550 (74.9%)	p-value
**VD supplementation**	**455 (62.0)**	**79 (42.9)**	**376 (68.4)**	**<0.001**
**Sex: female**	**551 (75.1)**	**106 (57.6)**	**445 (80.9)**	**<0.001**
**Age [years]**	**31 (24-39)**	**37 (28-49)**	**30 (23-37)**	**<0.001**
**Age > 65 years**	**11 (1.5)**	**9 (5)**	**2 (0.4)**	**<0.001**
**Diabetes duration [years]**	**12 (5-20)**	**14 (7-23)**	**12 (5-19)**	**0.04**
**Weight [kg]**	**68 (60-79)**	**73 (62-83)**	**67 (60-78)**	**<0.001**
**Height [m]**	**1.69 (1.64-1.74)**	**1.71 (1.65-1.77)**	**1.68 (1.64-1.73)**	**<0.001**
BMI [kg/m²]	23.9 (21.5-26.7)	24.1 (21.7-27.5)	23.9 (21.5-26.6)	0.29
Overweight (BMI 25-30 [kg/m²])	193 (26.3)	50 (27.2)	143 (26.0)	0.75
Obesity (BMI ≥ 30 [kg/m²])	83 (11.3)	24 (13.0)	59 (10.7)	0.39
**Living place: village**	**193 (26.3)**	**64 (34.8)**	**129 (23.5)**	**<0.01**
Living place: city < 50,000 citizens	186 (25.3)	53 (28.8)	133 (24.2)	0.21
**Living place: city > 50,000 citizens**	**355 (48.4)**	**67 (36.4)**	**288 (52.4)**	**<0.001**
**At least one diabetic complication**	**156 (21.3)**	**55 (29.9)**	**101 (18.4)**	**<0.001**
**Retinopathy**	**106 (14.4)**	**42 (22.8)**	**64 (11.6)**	**<0.001**
**Nephropathy**	**28 (3.8)**	**14 (7.6)**	**14 (2.5)**	**<0.01**
Neuropathy	87 (11.9)	26 (14.1)	61 (11.1)	0.27
**Diabetic Foot Syndrome**	**20 (2.7)**	**10 (5.4)**	**10 (1.8)**	**<0.01**
**Ischaemic Heart Disease**	**24 (3.3)**	**13 (7.1)**	**11 (2.0)**	**<0.001**
**Hypothyroidism**	**226 (30.8)**	**40 (21.7)**	**186 (33.8)**	**<0.01**
Coeliac disease	32 (4.4)	4 (2.2)	28 (5.1)	0.09
Asthma	39 (5.3)	7 (3.8)	32 (5.8)	0.29

VD – Vitamin D

b) influence of the respondent environment

variables	Total<br>n = 734 (100%)	Paper surveys<br>n =184 (25.1%)	Digital surveys<br>n = 550 (74.9%)	p-value
**Family doctor recommendation: YES**	**109 (14.9)**	**18 (9.8)**	**91 (16.5)**	**0.03**
**Medical specialist recommendation: YES**	**262 (35.7)**	**38 (20.7)**	**224 (40.7)**	**<0.001**
Pharmacist recommendation: YES	53 (7.2)	16 (8.7)	37 (6.7)	0.37
Relative recommendation: YES	190 (25.9)	44 (23.9)	146 (26.5)	0.48
Friend recommendation: YES	148 (20.2)	45 (24.4)	103 (18.7)	0.09
Knowledge acquired from Internet/media/books: YES	288 (39.2)	61 (33.2)	227 (41.3)	0.05
My relative supplements VD: YES	287 (39.1)	75 (40.8)	212 (38.5)	0.59
My friend supplements VD: YES	243 (33.1)	66 (35.9)	177 (32.2)	0.36
**Number of positive responses [n]**	**2 (1-3)**	**2 (0-3)**	**2 (1-3)**	**0.046**

Table S5.
Univariate logistic regression analysis. Dependent variable: Vitamin D supplementation.
Variables with p-value < 0.1 were included into multivariate logistic model.

Independent variable	OR [95%CI]	p-value
**Digital version of the survey**	**2.87 [2.04-4.05]**	**< 0.001**
**Sex: female**	**1.37 [0.98-1.93]**	**0.07**
Age	0.99 [0.98-1.00]	0.13
Diabetes duration	0.99 [0.98-1.01]	0.46
Overweight or obese [BMI ≥ 25 [kg/m²]]	1.05 [0.77-1.43]	0.76
**Living place: village**	**0.56 [0.40-0.79]**	**< 0.001**
At least one diabetic complication	1.01 [0.70-1.45]	0.96
Retinopathy	0.59 [0.39-0.89]	0.01
Nephropathy	0.60 [0.28-1.28]	0.19
Neuropathy	1.19 [0.74-1.90]	0.47
Diabetic Foot Syndrome	0.60 [0.25-1.47]	0.27
Ischaemic Heart Disease	1.87 [0.73-4.78]	0.19
**Hypothyroidism**	**1.75 [1.25-2.45]**	**< 0.01**
Coeliac disease	1.60 [0.73-3.50]	0.24
Asthma	1.10 [0.56-2.15]	0.78
**Family doctor recommendation: YES**	**5.47 [3.00-9.98]**	**< 0.001**
**Medical specialist recommendation: YES**	**12.32 [7.74-19.60]**	**< 0.001**
**Pharmacist recommendation: YES**	**2.20 [1.14-4.27]**	**0.02**
**Relative recommendation: YES**	**1.97 [1.37-2.83]**	**< 0.001**
Friend recommendation: YES	1.26 [0.86-1.84]	0.24
**Knowledge acquired from Internet / media / books: YES**	**3.56 [2.54-4.99]**	**< 0.001**
**My relative supplements VD: YES**	**1.69 [1.23-2.31]**	**< 0.01**
**My friend supplements VD: YES**	**2.00 [1.43-2.79]**	**< 0.001**

BMI – Body Mass Index; CI – Confidence Interval, OR – Odds Ratio, VD – Vitamin D

Datasets are available in Mendeley repository:

DOI: 10.17632/5dyy7xfykk.2

Kamiński, Mikołaj; Molenda, Magdalena; Banaś, Agnieszka; Uruska, Aleksandra; Zozulińska-Ziółkiewicz, Dorota (2019), “Determinants of Vitamin D supplementation among individuals with type 1 diabetes. ”, Mendeley Data, V2, doi: 10.17632/5dyy7xfykk.
